# ^18^F-FDG PET/CT imaging and PET-guided biopsy in evaluation and treatment decision in adrenal histoplasmosis

**DOI:** 10.1259/bjrcr.20150451

**Published:** 2016-07-28

**Authors:** Renjith Radhakrishnan Kalathoorakath, Aman Sharma, Ashwani Sood, Uma Nahar, Arun Kumar Reddy Gorla, Bhagwant Rai Mittal

**Affiliations:** ^1^Department of Nuclear Medicine, PGIMER, Chandigarh, India; ^2^Department of Internal Medicine, PGIMER, Chandigarh, India; ^3^Department of Histopathology, PGIMER, Chandigarh, India

## Abstract

Histoplasmosis is a rare opportunistic fungal infection. It is commonly seen in immunocompromised individuals from endemic areas. Adrenal glands are frequently involved in the disseminated disease. Here, we present the case of a retropositive patient with constitutional symptoms, where whole-body positron emission tomography/CT scan revealed intense ^18^F-fludeoxyglucose uptake in bulky adrenal glands, and subsequent positron emission tomography-guided biopsy helped in establishing the diagnosis of adrenal histoplasmosis.

## Summary

Histoplasmosis is a rare opportunistic fungal infection. It is commonly seen in immunocompromised individuals from the endemic areas. Adrenal glands are frequently involved in the disseminated disease. Here, we present the case of a retropositive patient with constitutional symptoms, where whole-body positron emission tomography (PET)/CT revealed intense ^18^F-fludeoxyglucose (^18^F-FDG) uptake in bulky adrenal glands, and subsequent PET-guided biopsy helped in establishing the diagnosis of adrenal histoplasmosis.

## Background

Histoplasmosis infection is caused by the dimorphic fungi *Histoplasma capsulatum*. Chronic pulmonary infection is the usual presentation in histoplasmosis; however, disseminated disease may involve the reticuloendothelial system, gastrointestinal tract, urinary tract, central nervous system, bone marrow and adrenal glands, in addition to the lungs. The patients usually present with non-specific features. Most of the cases are reported from endemic areas and in disseminated form.^1^

Adrenal gland involvement may be uni- or bilateral with variable features seen on imaging. Ultrasonography or CT-guided transabdominal fine needle aspiration cytology or biopsy is usually performed to establish the diagnosis. However, the authors in this case report highlight the importance of whole-body FDG PET/CT in localization of the pathology and directing the biopsy site from the most metabolic region to establish the correct diagnosis and treatment with favourable outcome.

## Clinical presentation

A 26-year-old male presented with persistent fever and occasional spikes of high-grade fever for the past 4 months; he had episodes of loose stool and vomiting for 2 months. He had complaints of bilateral upper limbs numbness and burning sensation in bilateral lower limbs, resulting in difficulty in walking. He also had loss of appetite and weight. Physical examination and ultrasonography of the abdomen revealed hepatosplenomegaly, abdominal lymphadenopathy and impaired sensation in bilateral lower limbs (up to the groin on the right and up to the mid-thigh on the left) and fingers of both hands. He was also found to be seropositive for human immunodeficiency virus (CD4 count of 38). Biochemical parameters were within normal limits. He was put on modified empirical antitubercular treatment owing to the development of transaminitis during the course of disease at an outside hospital. The patient was referred to our institution in view of no relief of his symptoms. On further evaluation, cytomegalovirus polymerase chain reaction (for cytomegalovirus) was found to be positive, although the serology for histopalsmosis was not performed owing to non-availability in our institution.

## Investigations/imaging findings

**Figure 1.**
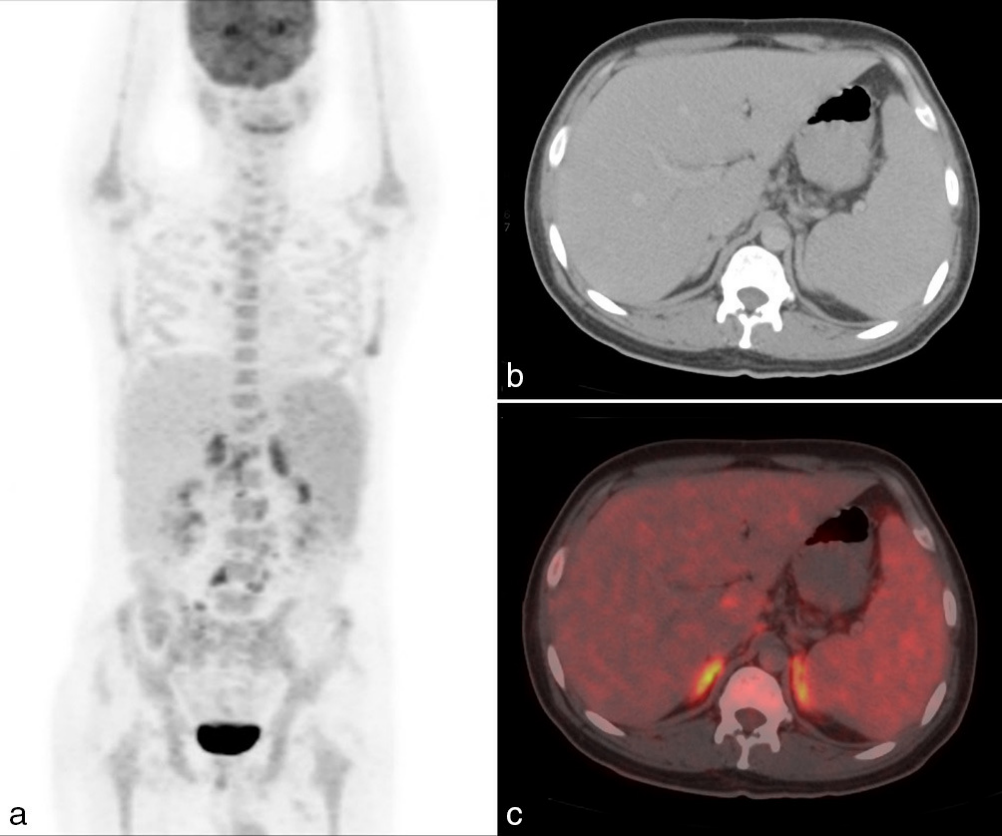
Maximal intensity projection image (a) showing intense fludeoxyglucose uptake in the bilateral suprarenal regions. Transaxial CT (b) and fused positron emission tomography/CT (c) images showing intense fludeoxyglucose uptake in the bilateral bulky adrenal glands.

**Figure 2.**
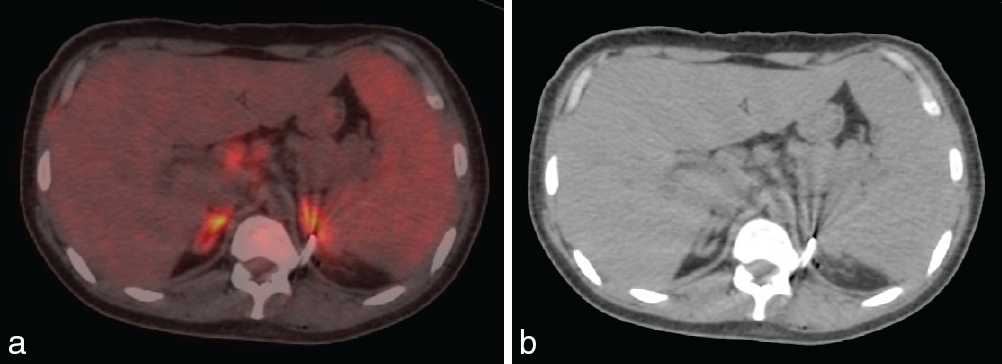
Fused transaxial positron emission tomography/CT (a) and CT (b) images showing the tip of the percutaneous biopsy needle precisely positioned in the left adrenal gland under robotic arm guidance.

**Figure 3.**
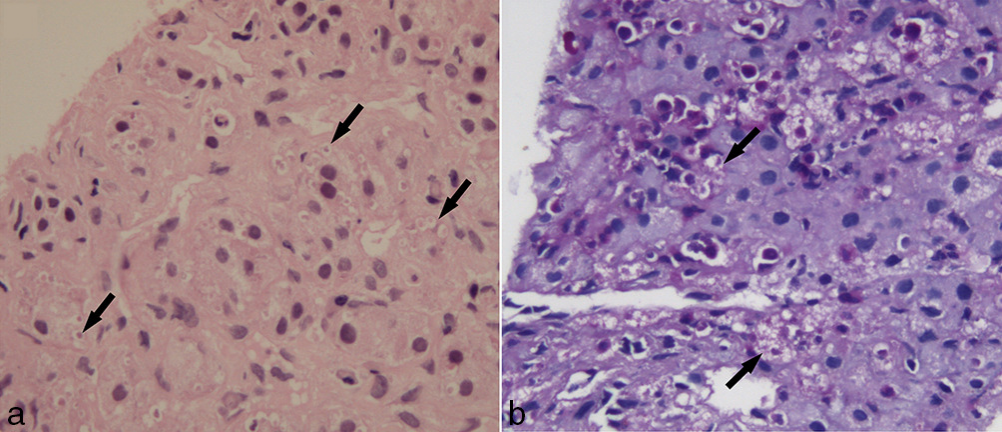
Microphotograph of the biopsy specimen in (a) haematoxylin and eosin (20×) staining and (b) periodic acid–Schiff (20×) staining showing infiltration of foamy histiocytes containing spores of histoplasma (arrows).

Whole-body ^18^F-FDG PET/CT showed intense FDG uptake in abdominal lymph nodes and bilateral bulky adrenal glands with evidence of hepatosplenomegaly (Figure 1). The patient underwent PET-guided biopsy from the FDG-avid bulky left adrenal gland (maximum standard uptake value of 7.9) (Figure 2). The adrenal gland was chosen as the site of biopsy, as the small-sized tracer-avid abdominal lymph nodes were in close vicinity of vascular structures. The automated robotic arm (ROBIO-EX, Perfint Healthcare Pvt. Ltd, Chennai, India) was used for navigating the needle after defining points of insertion on the skin surface and the target lesion in the digital imaging and communications in medicine images. A posterior paravertebral percutaneous route was chosen owing to the proximity of the lesion, ease of approach as well as the consideration of safe trajectory. Three fragments of adrenal tissue were obtained and the histopathology of two fragments showed focal polymorph-rich inflammatory infiltrate admixed with scattered foamy histiocytes and occasional giant cells. These foamy histiocytes showed increased small round to oval fungal elements with a halo corresponding to the morphology of histoplasma, which was highlighted by periodic acid–Schiff stain (Figure 3). One fragment was necrotic.

## Treatment and follow-up

The patient was put on appropriate antifungal treatment and showed significant improvement on clinical follow-up.

## Discussion

Histoplasmosis infection is usually subclinical or self-limiting in individuals with intact immunity; however, immunocompromised patients may have full-blown disseminated disease that can be fatal, particularly in human immunodeficiency virus-positive patients and transplant recipients. Disseminated histoplasmosis may involve the adrenal glands in addition to other organs. Unilateral adrenal gland involvement is more frequent.^3,4^ Isolated adrenal involvement with adrenal insufficiency as the presenting manifestation of histoplasmosis is even rarer; however, it should be considered as a possibility when clinical suspicion is high.^5^ Adrenal involvement with no clinical or biochemical evidence of adrenal insufficiency and without any feature of pulmonary involvement was noted in the index case.

The mean age at diagnosis of adrenal histoplasmosis in immunocompetent patients is usually high,^6^ although our patient was young and immunocompromised. Detection of bilateral adrenal masses on imaging in any patient often presents a management dilemma and may have differential diagnosis of neoplastic disorders, adrenal hyperplasia, infections such as tuberculosis, histoplasmosis, blastomycosis and adrenal haemorrhage, but imaging characteristics help in narrowing down the diagnosis.^7^ Definitive diagnosis requires demonstration of the organism by culture or histology. Radiologically, adrenal histoplasmosis is appreciated as a peripherally enhancing bilateral symmetrical enlargement of the glands with areas of relatively lower attenuation centrally attributed to necrosis/haemorrhage within the lesion. The contours of the glands are usually preserved. Similar CT features are also seen in tuberculosis, cryptococcosis and metastasis.^8^

^18^F-FDG PET/CT scan has also been extensively used to help in the diagnosis and assessment of the extent of the disease and monitoring the treatment response in adrenal histoplasmosis. Follow-up PET/CT scan has shown a significant decrease in tracer uptake after appropriate therapy.^9,10^ Umeoka et al^10^ showed intense tracer uptake in bilateral adrenal histoplasmosis on FDG PET study. Shah et al^11^ also reported intense FDG uptake in bilateral adrenal masses and the histopathological diagnosis of adrenal histoplasmosis based on CT-guided biopsy.^12^ Increased FDG uptake is also seen in adrenal tuberculosis, and differentiation between tuberculosis and histoplasmosis is particularly important in those parts of the world where both are endemic. Histopathological confirmation of the FDG-avid adrenal lesions is mandatory, as the treatment modalities in both entities are different.

Percutaneous adrenal biopsies under CT/ultrasound guidance are safe and well-established procedures in retrieving specimens from adrenal lesions for histopathological examination. However, Silverman et al^12^ in their study have reported a non-diagnostic rate of 14% for percutaneous adrenal biopsies, which might be attributed to sampling from necrotic or haemorrhagic areas. It is believed that percutaneous biopsy with ^18^F-FDG-PET guidance can resolve and reduce the issue of non-diagnostic samples with similar complication rates as that of CT-guided percutaneous biopsies. The robotic arm assists in precise targeting of the metabolically active area within a lesion, avoiding necrotic/haemorrhagic/non-viable areas. The enhanced precision associated with the procedure eliminates spatial errors and reduces the number of needle insertions. Metabolically active areas within the lesions (even the small lesions) are targeted, as was performed in the index case showing increased FDG uptake in both adrenals (left adrenal lesion: maximum standard uptake value 7.9) and tracer-avid abdominal nodes. Cerci et al^13^ showed the impact of FDG-PET guided biopsy in patients with cancer where 128 out of 130 FDG-positive lesions were successfully accessed under PET guidance. Among these, 23 patients with previous non-tumoral biopsy report were referred for PET-guided biopsy and 21 out of 23 lesions were found to be malignant.

Therefore, it is prudent to target the FDG-avid areas under PET imaging guidance during biopsy. The samples obtained were likely to be more representative of the actual pathology and help in reducing the false-negative and inconclusive results owing to necrotic and haemorrhagic areas. However, we believe this is the first case report where PET image-guided biopsy was performed in an adrenal lesion in which FDG PET/CT scan fused images were used for planning, as well as to confirm the needle position in obtaining the specimen from the most metabolic area.

## Learning points

1. The various inflammatory or infectious conditions in immunocompromised patients with adrenal involvement in an endemic area may have similar radiological presentation, and histopathological analysis is necessary to confirm the diagnosis.
2. Whole-body FDG PET/CT scan shows the most accessible site for biopsy and reveals other sites of disease involvement.
3. Within a lesion, PET/CT scan can guide sampling from the most viable area, avoiding necrotic/haemorrhagic area.
4. PET/CT-guided biopsy can reduce false-negative results.

## Consent

Informed consent to publish this case (including images and data) was obtained and is held on record.
